# Effects of Ammonia-Nitrogen-Reducing Biofilm on Stress Responses and Muscle Quality in Crucian Carp During Transportation

**DOI:** 10.3390/foods15071189

**Published:** 2026-04-01

**Authors:** Xianxian Zhang, Liangzi Zhang, Han Yang, Ling Peng, Ramy M. Khoder, Ru Liu, Juan You, Tao Yin

**Affiliations:** 1 College of Food Science and Technology, National R&D Branch Center for Conventional Freshwater Fish Processing (Wuhan), Huazhong Agricultural University, Wuhan 430070, China; 2 College of Food Science and Technology, Wuhan Business University, Wuhan 430056, China; 3 Faculty of Agriculture, Benha University, Moshtohor, Toukh 13736, Egypt

**Keywords:** ammonia-nitrogen-reducing biofilm (aquatic nitrifying bacteria biofilm media), Crucian carp, fish bag transport, stress response, muscle quality

## Abstract

This study evaluated the efficacy of ammonia-nitrogen-reducing biofilms (aquatic nitrifying bacteria biofilm media, a fixed-bed biofilm capable of simultaneous nitrification and denitrification) in mitigating water quality deterioration and transport-induced physiological stress in live-transported Crucian carp (*Carassius auratus*). In a simulated bag transport system, the application of the biofilm significantly decreased ammonia-nitrogen concentrations through enhanced nitrification, stabilized pH and dissolved oxygen dynamics, and suppressed nitrite accumulation. Correspondingly, biofilm-treated fish exhibited significantly reduced systemic stress responses, as evidenced by reduced serum cortisol, glucose, and lactate dehydrogenase concentrations, along with diminished histopathological changes in gill and liver tissues and preserved muscle fiber integrity. Regarding post-transport muscle quality, biofilm treatment delayed glycogen catabolism and lactate accumulation, maintained elevated muscle pH and water-holding capacity, reduced shear force decline, decelerated ATP hydrolysis and freshness degradation (K-value), and simultaneously suppressed lipid peroxidation and myonuclear apoptosis. These findings demonstrate that ammonia-nitrogen-reducing biofilms represent a viable biotechnological approach for maintaining water quality, mitigating stress-induced physiological disturbances, and preserving flesh quality during live fish transportation. This approach has significant potential for improving post-harvest outcomes in aquaculture logistics.

## 1. Introduction

Live transportation serves as a critical link in modern fishery distribution, enabling resource allocation across different regions to meet market demand. With the advancement of cold chains for fresh aquatic products and e-commerce, bagged live fish transport has emerged as a mainstream method for distributing high-value live fish. This approach offers flexibility, hygiene, suitability for long-distance small-batch shipments, and cost-effectiveness, particularly in the inland regions of developing countries [1]. However, the confined transport environment also leads to the accumulation of a series of severe stressors, posing a serious threat to fish health and muscle quality [2]. During transport, fish inevitably face environmental factors such as ammonia nitrogen [3], temperature [4], hypoxia [5], and overcrowding [6,7]. When subjected to adverse stimuli, fish mount intense stress responses to maintain their physiological equilibrium. This triggers energy metabolism disorders and redox system imbalances, ultimately reducing survival rates and degrading muscle quality [8,9], thereby diminishing both edibility and economic value.

Pre-transport measures, such as fasting to empty the digestive tract, adding salts, regulating dissolved oxygen and temperature, and using water conditioners, have been reported to maintain water quality stability during transport and reduce stressors [10]. Based on factors including fish physiology, body size, space requirements, transport duration, and temperature, rationally setting transport loading density is a key measure to effectively suppress water quality deterioration and reduce transport mortality [1,11]. The exogenous addition of substances such as ATP to water can effectively enhance the survival rates of aquatic animals under stressful conditions and alleviate oxidative stress [12]. Kamalam et al. [1] demonstrated in a study of transporting rainbow trout fry for 40 h in a plastic bag that a 72 h fasting period, optimized loading density, and clove oil sedation could achieve zero mortality post-transport. Additionally, adding 6 g/L salt significantly reduced non-ionic ammonia concentrations in the transport water. However, these measures essentially address ammonia nitrogen indirectly by lowering fish metabolism or adjusting water quality parameters and do not eliminate ammonia nitrogen directly from the transport system. The core of the biofilm method for ammonia nitrogen reduction lies in the utilization of nitrifying bacteria immobilized on carriers to oxidize ammonia nitrogen into nitrate [13]. By introducing high-efficiency microbial agents, the biological conversion process of ammonia nitrogen is accelerated, thereby effectively promoting nitrification and reducing ammonia nitrogen concentrations in water. Jiao et al. [14] achieved a 96.02% ammonia nitrogen removal rate by constructing a composite microbial consortium. Deng et al. [15] reported > 88% nitrogen removal efficiency using immobilized Pseudomonas sp. DM02 in aquaculture wastewater. Research indicates that in situ biofilm systems constructed using artificial substrates rapidly convert ammonia nitrogen into less toxic nitrates, thereby stabilizing water quality [16]. Moreover, biofilm systems not only improve aquatic environments but also alleviate stress in farmed organisms by providing shelter and natural food sources, thereby enhancing their health status [17]. Although biofilm technology is currently used in aquaculture, its effectiveness during live fish transportation remains unclear.

Therefore, this study used the Crucian carp (*Carassius auratus*) as the research subject and incorporated ammonia-nitrogen-reducing biofilms into a simulated transport system to investigate their effects on water quality, stress indicators in the Crucian carp, and muscle quality. This study aims to provide new technological approaches and theoretical foundations for the transportation of live fish. The ammonia-nitrogen-reducing biofilm technology evaluated in this study offers a potential solution that could improve survival rates, maintain muscle quality, and reduce economic losses in commercial live fish transport operations.

## 2. Materials and Methods

### 2.1. Materials

The Crucian carp used in this experiment were sourced from the Wuhan Zhihui Nongyan Aquaculture Base in Wuhan City, Hubei Province, China. The average body weight of the Crucian carp was 250 ± 50 g, with a body length of 30 ± 5 cm. The water used in the experiment was aerated tap water. The live fish transport bags were made of polyethylene material purchased from Xinsuda Plastic Products Factory in Baoding, Hebei Province, China (40 cm × 25 cm, with a thickness of 18 mil). The ammonia-nitrogen-reducing biofilm’s (K5, Wuhan Water Kingdom Environmental Technology Co., Ltd., Wuhan, China) core technology is based on the patent “A Composite Bacterial Agent of Nitrosomonas and Nitrifying Bacteria-Denitrifying Bacteria, Production Method, and Application” (CN 105274029 A). This biofilm, classified as an immobilized composite functional biofilm, immobilizes the composite bacterial agent and primarily includes the following functional strains:

(1) *Nitrosomonas europaica* oxidizes ammonia nitrogen (NH_4_^+^) in water to nitrite (NO_2_^−^);
(2) *Nitrospira Y3-2* further oxidizes nitrite to nitrate (NO_3_^−^);
(3) *Pseudomonas schiffii* performs denitrification under low-oxygen conditions, converting nitrate to nitrogen gas (N_2_), thereby removing nitrogen from water bodies.

The ratio of the three strains in the composite bacterial agent is: (1–5) × 10^8^ CFU/mL:(1–5) × 10^8^ CFU/mL:(1–10) × 10^8^ CFU/mL. The biofilm is a piece-like carrier, each piece measuring approximately 3 cm × 3 cm and containing a total live bacteria load ≥ 1 × 10^9^ CFU/piece.

### 2.2. Fish Transportation Protocol

All animal standard operating procedures were approved by the Animal Care and Use Committee of Huazhong Agricultural University and performed in accordance with the Guidelines for Care and Use of Laboratory Animals of Huazhong Agricultural University (HZAUFI-2024-0042).

The transportation protocol was adopted from Yang et al. [18] with minor modifications. Freshly caught and disinfected Crucian carp were temporarily held in prepared tanks for 2 days. Subsequently, similarly sized, healthy, and vigorous carp were randomly allocated for two sequential experiments. Before the commencement of formal trials, biofilm dosage screening must be conducted. Fish were randomly divided into three groups of 10 individuals each bag, corresponding to biofilm loads of 0, 2 and 5 per bag. Fish-to-water ratio was 1:3 (*w*/*v*). Bags were sealed, oxygenated, and placed on a simulated transport platform (JHY-5024, Jinhuayuan Technology Co., Ltd., Xiamen, China) for up to 72 h. Survival rates were recorded at 12 h intervals, and water quality parameters (pH, dissolved oxygen, ammonia-nitrogen, nitrite) were measured at each time point. Based on the survival rate and water quality results, the optimal biofilm dosage (3 pieces per bag) was selected for subsequent experiments (Section 3.1 and Section 3.2). Using the optimal biofilm dosage determined before, two groups were established: a control group (CG, without biofilm) and a treatment group (TG, with 3 pieces of biofilm per bag). Each group consisted of 30 fish, randomly divided into three groups of 10 individuals each bag, from which three fish were randomly sampled from three different bags at each time point for serum biochemical, oxidative stress, and muscle quality analyses. Gill, liver, and muscle tissues were collected at 48 h for histological examination. The transport temperature was maintained at 25 ± 0.5 °C throughout the 72 h simulated transport using the temperature-controlled system of the simulated transport platform. This temperature was selected based on preliminary optimization experiments (Supplementary Table S1), which demonstrated that 25 °C yielded the highest ammonia-nitrogen reduction efficiency. Water temperature was monitored at each sampling time point and remained stable throughout all experiments. The fish bags were filled with aerated tap water, ammonia-nitrogen-reducing biofilms, and sodium percarbonate. After sealing with a sealing machine (300/8 mm, Kangming Packaging Equipment Co., Ltd., Suzhou, China), the bags were oxygenated to achieve an initial internal pressure of approximately 12 kPa. The fish were then placed in foam-insulated boxes with ice packs equivalent to 50% of the total weight of the fish and water.

During transport, blood physiological and biochemical indicators, oxidative stress indicators, and muscle quality indicators were measured at 0, 12, 24, 36, and 48 h. Changes in the gill, liver, and muscle tissue structures were assessed after 48 h of live transport. Before serum collection, the MS-222 solution (120 mg/L) was injected into the transport bags to minimize stress responses in Crucian carp. Once the fish were under deep anesthesia, they were removed for subsequent procedures [19]. A 5 mL syringe was immediately inserted into the main vertebral column, approximately 3 cm from the tail, to draw blood, which was collected in a 1.5 mL centrifuge tube. After removing the scales, heads, and viscera, the fish were split along the spine into two halves. The dorsal muscle located below the dorsal fin and above the lateral line was used. The liver and gills were simultaneously dissected and removed. After the blood was allowed to stand for 1 h, it was centrifuged at 4000 rpm for 10 min. The supernatant was collected into a 2 mL cryovial, rapidly frozen in liquid nitrogen, and transferred to a −80 °C ultra-low-temperature freezer (Haier Bio-Medical Co., Ltd., Qingdao, China) for the determination of blood biochemical parameters and blood oxidative stress markers. The liver was rinsed with PBS, blotted dry with filter paper, and cut into multiple chunks of approximately the size of a soybean. These were placed in a 5 mL round-bottom centrifuge tube and immersed in 4% (*w*/*v*) paraformaldehyde for fixation for at least 24 h, followed by histological examination. The gills were rinsed with PBS, dried with filter paper, cut into multiple strips no wider than 0.5 cm, placed in 50 mL round-bottom centrifuge tubes, and then handle it like the liver. For larger gill tissue sections, switch to the EDTA decalcification solution, replacing the solution every three days until the tissue softens. After determining the pH of the fish meat, it was cut into 20 mm × 20 mm × 10 mm cubes using a scalpel. Color, drip loss, and shear force were measured immediately. For muscle tissue structure and apoptosis staining, fish meat was cut into thin slices the size of soybeans, placed in 50 mL round-bottom centrifuge tubes, and immersed in 4% (*w*/*v*) paraformaldehyde for fixation for at least 24 h. The remaining samples were portioned into self-sealing bags and stored at −80 °C within 0.5 h post-mortem for analysis of basic nutritional composition, lactic acid, glycogen, ATP, and its metabolic byproducts, muscle redox indicators, and reactive oxygen species staining.

### 2.3. Determination of Water Environmental Parameters

The simulated transport time lasted up to 72 h, with measurements taken every 12 h to determine the pH, ammonia nitrogen concentration, dissolved oxygen, and nitrite content at each time point. The pH was measured using a pH meter (Model 818, Smart Sensor Co., Ltd., Hong Kong, China). Ammonia nitrogen concentration was determined with an ammonia nitrogen analyzer (Model YC 7200-N, Analytic Technology Co., Ltd., Shenzhen, China). Dissolved oxygen was measured using a dissolved oxygen meter (Model AR 8210, Smart Sensor Co., Ltd., Hong Kong, China). Nitrite content was measured using an enzyme-linked immunosorbent assay reader (Multiskan SkyHigh, Thermo Fisher Scientific, Waltham, MA, USA). Each individual bag served as the experimental unit. Measurements were taken from three replicate bags per treatment group at each time point, and the mean value was used for statistical analysis. Three replicates were carried out for each group.

### 2.4. Determination of Survival Rate

The simulated transport duration reached 72 h, and survival rates were calculated at 12 h intervals. In this experiment, Crucian carp exhibiting signs such as gasping at the surface or rolling onto their backs, showing no response to repeated stimulation, or displaying systemic hemorrhaging and extremely irregular respiration, followed by complete cessation of respiration within 5–10 min, were deemed deceased [20]. Three replicates were carried out for each group.

The calculation formula is as follows:

(1) $$w_{t}\left(\%\right)=\frac{n_{t}}{10}\times 100\%$$

where w_t_ is the survival rate of each group at different times; n_t_ is the survival quantity of each group at different times.

### 2.5. Determination of Serum Biochemical Indicators

Serum biochemical markers were measured using enzyme-linked immunosorbent assay (ELISA) and fully automated biochemical analysis (Chemray 240, Shenzhen Leidu Life Sciences Co., Ltd., Shenzhen, China), using kits from Shanghai Jingkang (Shanghai, China) and Beijing Solarbio Technology Co., Ltd. (Beijing, China). The assayed markers included cortisol (COR), glucose (GLU), lactate dehydrogenase (LDH), alkaline phosphatase (AKP), aspartate aminotransferase (AST), alanine aminotransferase (ALT), UREA, and creatinine (CREA). Three replicates were carried out for each group.

### 2.6. Determination of Redox Indications in Serum and Muscle

The levels of oxidative stress markers in fish serum and muscle tissue were measured using a kit (Beijing Solarbio Science & Technology Co., Ltd., Beijing, China). The markers assessed included malondialdehyde (MDA), total antioxidant capacity (T-AOC), and the activities of superoxide dismutase (SOD), catalase (CAT), and glutathione peroxidase (GPx). Three replicates were carried out for each group.

### 2.7. Observation of Gill, Liver, and Muscle Tissue Structures

Using forceps, carefully remove the gills, liver, and muscle tissues that have been fixed for 24 h. The surface moisture was blotted using filter paper. The samples were immersed in xylene twice for 20 min each, then in anhydrous ethanol twice for 5 min each, followed by 5 min in 75% (*v*/*v*) ethanol. Finally, the samples were rinsed with tap water to complete the “paraffin section dewaxing to water’’ procedure. The sections were placed in hematoxylin stain for 3–5 min, rinsed with tap water between each step. The sections were dehydrated sequentially in 85% (*v*/*v*) and 95% (*v*/*v*) ethanol for 5 min each, and then immersed in eosin solution for 5 min to complete eosin staining. The sections were successively immersed in anhydrous ethanol (5 min each) and two immersions in xylene (5 min each). At this point, the sections became nearly transparent and were mounted with neutral resin. The samples were examined under an optical microscope (Eclipse E100, Nikon Corporation, Tokyo, Japan), and panoramic scanning was performed using an imaging system (DS-U3, Nikon Corporation, Tokyo, Japan).

Reactive oxygen species (ROS) levels in muscle tissue were determined after dihydroethidium (DHE) staining, Mitotracker staining DAPI counterstaining. Additionally, apoptosis levels in muscle tissue were evaluated following TUNEL staining. Three replicates were carried out for each group.

### 2.8. Determination of Muscle Quality-Related Indicators

#### 2.8.1. Shear Force

Shear force measurements were performed using a physical property analyzer equipped with a blade (RHEO TEX SD-700, Sun Scientific Corporation, Tokyo, Japan). As each muscle segment of the fish consists of longitudinally arranged fibers, shear force was applied along the longitudinal direction of the muscle fibers during measurement [21]. Under optimal viability conditions, three fish were randomly selected at each time point for analysis. The dorsal muscle of each fish was cut into 20 mm × 20 mm × 10 mm cubes, and measurements were performed on three samples per time point for each group. Muscle samples were cut using an XL 1155 blade (Xie Li Electronic Technology Group Co., Ltd., Nanjing, China), and the shear force values were expressed in grams. Three replicates were carried out for each group.

#### 2.8.2. Drip Loss

Drip loss was determined using the hanging weight method, as described by Subbaiah et al. [22]. After survival preservation, the dorsal muscle was cut into small cubes measuring 20 mm × 20 mm × 10 mm and weighed individually. The weighed fresh dorsal muscle was suspended for storage in a 4 °C refrigerator (BCD-272 WDPD, Haier Smart Home Co., Ltd., Qingdao, China), and the muscle weight was measured at different time points. Three replicates were carried out for each group. The drip loss was calculated using the following formula:

(2) $$Drip\;loss\left(\%\right)=\frac{M1-M2}{M1}\times 100\%$$

where M1 is the pre-storage meat weight; M2 is the post-storage meat weight.

#### 2.8.3. Muscle Glycogen, Lactate, pH

Glycogen and lactate levels were measured using kits (Beijing Solarbio Technology Co., Ltd., Beijing, China). The pH was measured using a pH meter (Testo 205, Testo Instruments International Trading Co., Ltd., Shanghai, China). For each fish, measurements were taken at two locations on the dorsal muscle, near the head and tail. After the reading stabilized and an audible signal was emitted, the displayed value represented the pH of the dorsal muscle. Approximately 10 measurements were recorded for each group.

#### 2.8.4. Color

A handheld color difference meter (CR-400, Konica Minolta, Tokyo, Japan) calibrated against a standard white plate was used to test each group, with at least 10 samples measured for each group. The recorded values included lightness (L*), red/green (a*), and yellow/blue (b*) values. Values of a* > 0, a* < 0, b* > 0, and b* < 0 indicate reddish, greenish, yellowish, and bluish samples, respectively. The whiteness value W was calculated using the following formula:

The whiteness value W calculation formula is as follows:

(3) $$W=100-\sqrt{{\left(100-L^{*}\right)}^{2}+{\left(a^{*}\right)}^{2}+{\left(b^{*}\right)}^{2}}$$

#### 2.8.5. ATP and Metabolically Related Products

Following the method described by Peng et al. [20], the contents of hyperxanthine (Hx), inosine (HxR), inosinic acid (IMP), 5′-adenosine monophosphate (AMP), 5′-adenosine diphosphate (ADP), and 5′-adenosine triphosphate (ATP) were determined using high-performance liquid chromatography (HPLC, Agilent 1260, Agilent Corporation, Santa Clara, CA, USA). Collected dorsal muscle samples were placed in centrifuge tubes. Then, 20 mL of pre-chilled 5% perchloric acid solution was added. The mixture was homogenized twice using a homogenizer (KZ-III-FP, Wuhan Saivell Biotechnology Co., Ltd., Wuhan, China) at 10,000 rpm for 20 s each time. The homogenized blade was rinsed with 10 mL of the same perchloric acid solution. The rinse solution was combined with the centrifuge tube contents, centrifuged (4 °C, 10,000 rpm, 10 min), and the supernatant was aspirated. The pellet was washed with 10 mL of the same concentration of perchloric acid, centrifuged, and the two supernatants were combined, and the pH was adjusted to 6.4 using 10 mol/L and 1 mol/L NaOH. The solution was diluted to 50 mL with ultrapure water, and 1 mL was filtered through a 0.45 μm membrane filter. The filtrate was then subjected to HPLC analysis. HPLC Conditions were as follows: column: XBridge BEHC 18 Column (5 μm, 4.6 mm × 250 mm); column temperature: room temperature; injection volume: 10 μL; mobile phase: 0.02 mol/L phosphate buffer (pH = 6.4); flow rate: 0.7 mL/min. The freshness value K was calculated as the percentage of HxR and Hx relative to the total ATP-associated metabolic products. Three replicates were carried out for each group.

The freshness value K (%) was calculated as follows:

(4) $$K=\frac{HxR+Hx}{ATP+ADP+AMP+IMP+HxR+Hx}\times 100\%$$

### 2.9. Statistical Analysis

All data are presented as the mean ± standard deviation (SD). The number of independent replicates (*n*) for each experiment is specified in the corresponding figure legend or table footnote. Data were analyzed using SPSS version 22.0 (IBM Corp., Armonk, NY, USA). Univariate analysis of variance (ANOVA) was performed using Duncan’s multiple range test for post hoc comparisons. Statistical significance was set at a 95% confidence level (*p* < 0.05). Graphs were generated using GraphPad Prism 9.4 (GraphPad Software, San Diego, CA, USA).

## 3. Results

### 3.1. Effect of Ammonia-Nitrogen-Reducing Biofilm on Crucian Carp Survival Rate During Transportation

**Figure 1:**
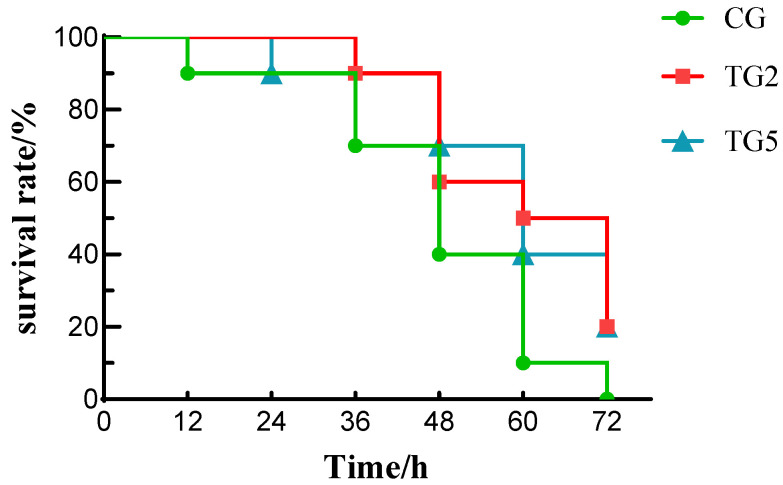
Changes in the survival rate of Crucian carp during fish transportation. Note: Values are represented mean of three independent replicates (*n* = 3) per group. CG: control group (0 piece); TG2: treatment with 2 biofilm pieces; TG5: treatment with 5 biofilm pieces. The same as below.

To determine the effective dosage of the ammonia-nitrogen-reducing biofilm, we first evaluated the effects of three loads (0, 2 and 5 per bag) on water quality and fish survival during 72 h simulated transport. As shown in Figure 1, when no ammonia-nitrogen-reducing biofilm was applied, the survival rate after 12 h of live transport decreased to 90%, whereas the survival rates in the other two groups remained at 100%. After 60 h of live transport, the survival rate for the group with zero ammonia-nitrogen-reducing biofilms was 10%, while the groups with two and five biofilms had survival rates of 50% and 40%, respectively. After 72 h of live transport, the survival rate of the group with zero biofilms was 0%, whereas the other two groups had survival rates of 20%.

**Figure 2:**
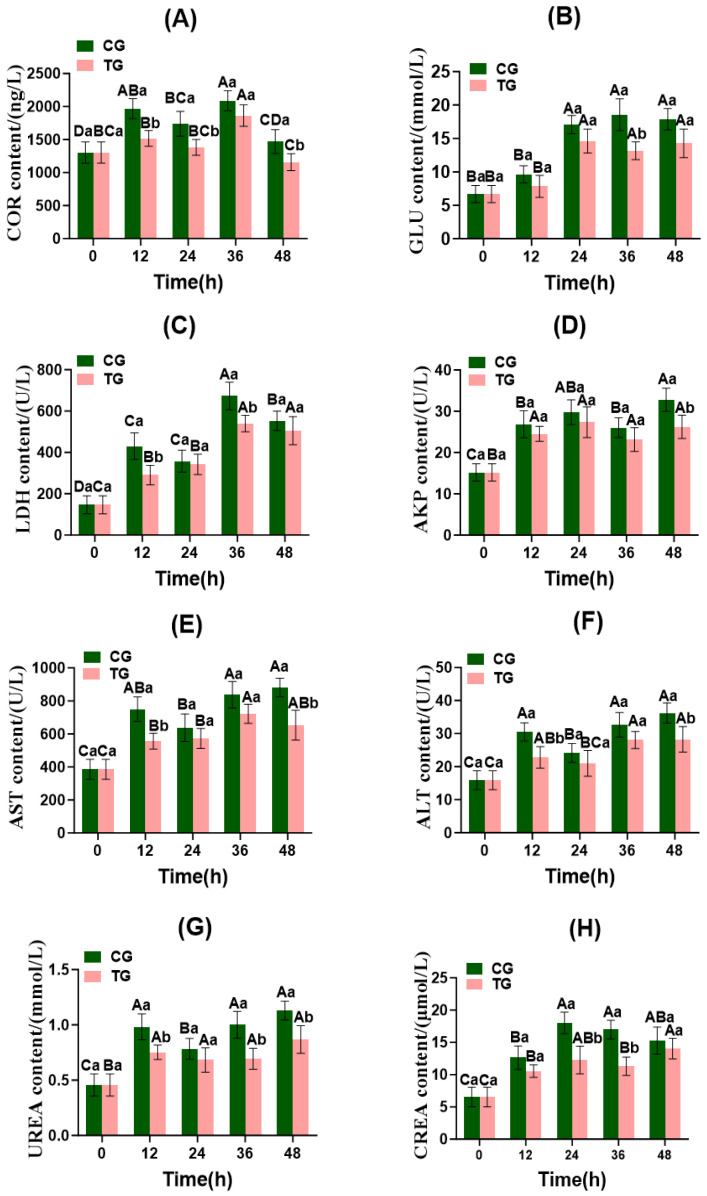
Changes in serum biochemical indices of Crucian carp during transportation in fish bags. CG: control group; TG: treatment group. (**A**) COR: cortisol, (**B**) GLU: glucose, (**C**) LDH: lactate dehydrogenase, (**D**) AKP: alkaline phosphatase, (**E**) AST: aspartate aminotransferase, (**F**) ALT: alanine aminotransferase, (**G**) UREA: urea, (**H**) CREA: creatinine. Note: Values are represented mean ± SD of three independent replicates (*n* = 3) per group. Different capital letters indicate significant differences within the same group at different time points (*p* < 0.05), and different lowercase letters indicate significant differences among different groups at the same time point (*p* < 0.05).

During live transport, fish in groups with added ammonia-nitrogen-reducing biofilms exhibited higher survival rates than those without this treatment. This was attributed to the biofilm nitrification process, which reduces ammonia levels in the transport bags (Table 1) and mitigates fish stress responses (Figure 2). However, increasing the biofilm load beyond two pieces decreased the survival rate at 60 h. This may occur because higher ammonia-nitrogen-reducing biofilm loading increases the oxygen consumption of nitrifying bacteria, competing with fish respiration for oxygen. Studies have shown that in low-oxygen environments, fish-excreted ammonia nitrogen cannot be effectively converted, and its toxicity increases as dissolved oxygen levels decrease, resulting in reduced survival rates among fish [23,24]. In addition, when the ammonia-nitrogen-reducing biofilm load is high, the concentration of nitrite, a product of the biofilm’s conversion of ammonia nitrogen and other substances through nitrification, also gradually increases (Table 1). Elevated nitrite levels in water can compromise fish immunity, adversely affecting survival rates [25].

### 3.2. Effects of Different Loading Levels on Water Quality Parameters During Fish Transportation

As shown in Table 1, the water pH exhibited a decreasing trend over time and with reduced ammonia-nitrogen-degrading biofilm loading levels. During the later stages of live transport, significant differences in water pH were observed among the three groups with different biofilm loading levels (*p* < 0.05). In CG, TG2, and TG5, respectively, the dissolved oxygen content in the water decreased from 5.80 mg/L at the initial 0 h mark to 4.63, 4.23, and 3.77 mg/L at 72 h. At 72 h of live transport, ammonia nitrogen concentrations in the TG2 and TG5 biofilms were 37.08 mg/L and 35.69 mg/L, respectively, while the concentration in the CG was 46.64 mg/L (*p* < 0.05). At 48 h of live transport, nitrite concentrations increased with higher biofilm loading, reaching 0.32 mg/L, 0.50 mg/L, and 0.58 mg/L in the CG, TG2 and TG5.

Dissolved oxygen levels in fish bags increased during the early transport phase, likely due to the slow release of oxygen from sodium percarbonate into the water. During the later stages of live transport, the dissolved oxygen levels gradually decreased. This was attributed to oxygen consumption from nitrification by the ammonia-nitrogen-reducing biofilm and fish respiration, leading to significant differences in the dissolved oxygen levels among different biofilm loading rates (*p* < 0.05). The nitrite concentrations in the water samples differed significantly (*p* < 0.05) at the same transport time point but under different ammonia-nitrogen-reducing biofilm loading conditions. During live transport in fish bags, ammonia-nitrogen-reducing biofilms effectively reduced water ammonia-nitrogen levels. The reduction in ammonia-nitrogen achieved by the biofilm was accompanied by an increase in nitrite and a decline in dissolved oxygen, particularly at higher biofilm loadings (5 pieces). This trade-off is inherent to the nitrification process: ammonia-oxidizing bacteria (AOB) convert ammonia to nitrite, and nitrite-oxidizing bacteria (NOB) further oxidize nitrite to nitrate, both steps consuming oxygen [26]. Under oxygen-limiting conditions, NOB are more sensitive than AOB, leading to transient nitrite accumulation [27]. The increased oxygen demand from higher biofilm loads exacerbates this imbalance, explaining the observed nitrite build-up and dissolved oxygen (DO) decline. When oxygen is abundant, nitrite is converted into nitrate [26].

### 3.3. Changes in Biochemical Indicators of Crucian Carp Serum During Fish Transportation

#### 3.3.1. Hormonal Indicators

Based on the dosage screening results (Section 3.1 and Section 3.2), the 3 pieces per bag dosage was selected as optimal for detailed analysis of stress responses and muscle quality. Therefore, in the following sections, CG refers to the control group (0 piece) and TG refers to the treatment group with 3 biofilm pieces per bag. As shown in Figure 2A, COR levels in the CG and TG differed significantly at 12, 24, and 48 h of live transport (*p* < 0.05). Both the CG and TG exhibited an overall trend of initial increase, followed by a decrease in COR levels, reaching peak values of 2086.77 ng/L and 1861.75 ng/L at 36 h of live transport, respectively. At 48 h of live transport, the COR levels were 1156.13 ng/L in the TG and 1468.80 ng/L in the CG.

In fish, stress activates the hypothalamic–pituitary–adrenal axis, leading to elevated blood cortisol levels [28]. At 12 h of live transport, COR levels in both the CG and TG rapidly increased, indicating that changes in the external environment during simulated transport triggered stress responses in the fish, causing them to secrete large amounts of cortisol to regulate glucose metabolism. After 24 h of live transport, COR levels in both the CG and TG declined, indicating gradual adaptation to the transport conditions. At 36 h, the COR levels increased again in response to the accumulated ammonia nitrogen stress (Table 1). After 48 h, the COR levels in both groups decreased markedly, possibly due to weakened vital functions, reduced metabolic activity, and impaired adrenal gland function in crucian carp under prolonged external stress, leading to decreased cortisol secretion. The COR levels in the CG were generally higher than those in the TG, indicating that the ammonia-nitrogen-reducing biofilm effectively reduced ammonia nitrogen levels in the water through nitrification, thereby mitigating stress responses in crucian carp.

#### 3.3.2. Energy Metabolism Indicators

As shown in Figure 2B,C, the GLU content in the CG exhibited a trend of initial increase followed by a decrease, the GLU content in the TG showed a pattern of initial increase, subsequent decrease, and then another increase. The CG reached a peak GLU level of 18.55 mmol/L at 36 h of live transport, and the TG reached a peak GLU level of 14.61 mmol/L at 24 h. Both CG and TG exhibited minimum GLU levels at 12 h of live transport, with significant differences observed at 36 h (*p* < 0.05). For LDH activity, significant differences were observed between the CG and TG at both 12 h and 36 h (*p* < 0.05). LDH activity peaked at 36 h in both groups, with minimum values recorded at 24 h in the CG and at 12 h in the TG.

During the early phase of live transport, the LDH levels in the TG gradually increased. This is attributed to stress-induced damage to the liver and myocardium resulting from altered environmental conditions, leading to elevated activities of related enzymes [29]. Conversely, LDH activity in the CG showed a decreasing trend, possibly due to the reduced metabolic activity caused by more severe stress in these fish. During the late phase of live transport, both the CG and TG exhibited decreased GLU and LDH levels. This is likely due to the high ammonia nitrogen concentrations in the water, which significantly impact fish physiology and metabolism, leading to weakened metabolic activity, a gradual reduction in enzyme activity, and inhibition of gluconeogenesis [30]. Throughout the live transport process, the GLU and LDH levels in the CG consistently exceeded those in the TG. This indicates that the TG Crucian carp exhibited a more stable physiological state, with the ammonia-nitrogen-reducing biofilm effectively lowering ammonia-nitrogen concentrations in the water (Table 1).

#### 3.3.3. Liver Function Indicators (AKP, AST, and ALT)

As shown in Figure 2D–F, the activities of AKP, AST, and ALT remained significantly higher in the CG than in the TG, indicating that the ammonia-nitrogen-reducing biofilm mitigated liver damage effectively. During the 12 h live transport, AKP activity increased markedly in both the TG and CG, indicating that high ammonia nitrogen concentrations can cause cellular damage within a short timeframe, leading to the release of intracellular AKP and a rapid elevation in blood AKP activity [31,32]. After 36 h of live transport, AKP activity decreased in both the CG and TG, indicating that the Crucian carp gradually adapted to the transport environment by regulating their immune systems and achieving a new physiological equilibrium. AST and ALT activities in both the CG and TG progressively increased with prolonged transport duration. This may be attributed to gradually rising ammonia nitrogen concentrations within the bags, impairing mitochondrial function and exacerbating the permeability of both plasma and mitochondrial membranes, thereby sustaining elevated enzyme activities [33]. Research has confirmed that ammonia exposure significantly elevates AST and ALT activity in liver tissue, consistent with the pathological process of increased permeability in hepatocyte and mitochondrial membranes [34]. These findings align with the changes in AST and ALT activities observed in this study. Throughout live transport, AKP, AST, and ALT activities were consistently higher in the CG than in the TG, further confirming that the ammonia-nitrogen-reducing biofilm effectively mitigated liver damage and thereby alleviated stress responses in Crucian carp.

#### 3.3.4. Renal Function Indicators (UREA and CREA)

As shown in Figure 2G,H, significant differences in UREA levels were observed between the CG and TG at 12, 36, and 48 h of live transport (*p* < 0.05). As the live transport duration increased, the UREA levels in both the CG and TG exhibited an overall pattern of initial rise, subsequent decline, and then another rise. CREA levels in the CG and TG showed significant differences at 24 and 36 h (*p* < 0.05). In the CG, CREA levels followed an initial rise, followed by a decline; in the TG, they showed an initial rise, followed by a decline, and then another increase.

Under ammonia nitrogen stress, fish renal excretory function is suppressed, leading to the accumulation of UREA and CREA in the blood. During live transport, UREA levels gradually increased in both the CG and TG, indicating that fish mitigate the physiological toxicity of ammonia nitrogen by enhancing urea synthetic pathways. However, impaired excretion results in urea accumulation in the blood. After 36 h of live transport, CREA levels decreased in both the CG and TG, indicating that fish gradually adapt to stressful environments by regulating metabolic mechanisms to cope with external pressures. CREA and UREA levels in the CG were generally higher than those in the TG, suggesting that the ammonia-nitrogen-reducing biofilm effectively mitigates stress responses in TG fish through nitrification, thereby reducing damage to kidney and gill tissues. Under cold stress, serum CREA levels in *Siganus fuscescens* (rabbitfish) exhibited an initial increase, followed by a decrease, potentially related to impaired renal excretory function [35], which aligns with the CREA concentration changes observed in the present experiment.

### 3.4. Changes in Reducer–Oxidizer Indicators in Crucian Carp Serum During Fish Transportation

**Figure 3:**
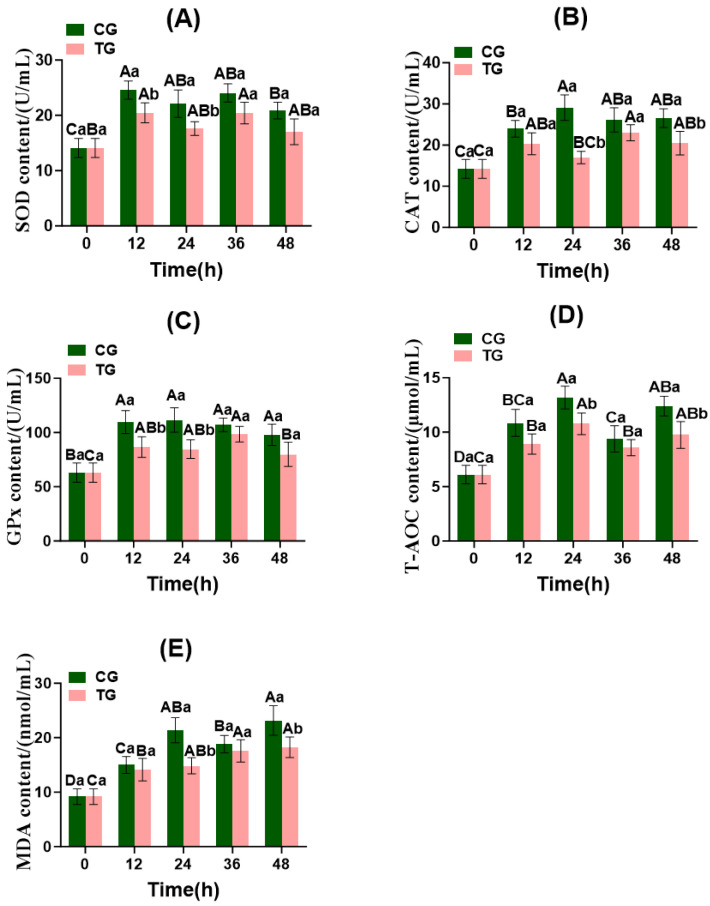
Changes in redox indexes of serum in Crucian carp during transportation in fish bags. CG: control group; TG: treatment group. (**A**) SOD, superoxide dismutase; (**B**) CAT, catalase; (**C**) GPx, glutathione peroxidase; (**D**) T-AOC, total antioxidant capacity; (**E**) MDA, malondialdehyde. Note: Values are represented mean ± SD of three independent replicates (*n* = 3) per group. Different capital letters indicate significant differences within the same group at different time points (*p* < 0.05), and different lowercase letters indicate significant differences among different groups at the same time point (*p* < 0.05).

As shown in Figure 3, SOD and GPx levels in the CG and TG differed significantly at 12 and 24 h post-transport (*p* < 0.05). CAT content in both the CG and TG showed significant differences at 24 and 48 h post-transport (*p* < 0.05), exhibiting an overall trend of initial increase followed by a decrease. Both the CG and TG exhibited a trend of initial increase, followed by a decrease, and then an increase in T-AOC. Significant differences in T-AOC were observed between the CG and TG at 24 and 48 h post-transport (*p* < 0.05). MDA levels in both the TG and CG showed an overall increasing trend, with significant differences between the CG and TG at 24 and 48 h of live transport (*p* < 0.05).

The CG generally exhibited higher antioxidant enzyme activity than the TG, while the CG had higher MDA levels. This indicates that the TG supplemented with the ammonia-nitrogen-reducing biofilm effectively alleviated oxidative stress in Crucian carp by reducing ammonia-nitrogen concentration and minimizing nitrite accumulation (Section 3.2). Under higher ammonia-nitrogen stress, the CG fish experienced a stronger initial ROS, which triggered a compensatory upregulation of antioxidant enzymes (SOD, CAT, GPx) to counteract oxidative damage [36]. However, as transport time extended, the persistent ROS production overwhelmed the antioxidant defense system, leading to enzyme inactivation and accumulation of lipid peroxidation products (MDA) [37]. In contrast, the TG experienced lower ammonia stress, resulting in a more balanced redox state with lower enzyme induction and less oxidative damage. During the early phase of live transport, SOD, CAT, and GPx levels increased in both the CG and TG, indicating rapid free radical accumulation in stressed Crucian carp. The body counteracts this by elevating antioxidant enzyme activity to eliminate excess radicals, thereby safeguarding normal metabolism and vital functions. In the late phase of live transport, SOD, CAT, and GPx activities gradually declined in both the CG and TG. This may result from excessively high ammonia nitrogen concentrations within the fish bags and the accumulation of free radicals, which impairs the body’s ability to scavenge these radicals. Consequently, gill and liver damage disrupts normal gas exchange functions [38]. Jin et al. [39] observed that SOD, CAT, and GPx activities all exhibited a pattern of an initial increase followed by a decline. This finding aligns with the observed trends in SOD, CAT, and GPx activities in the present study. During live fish transport, elevated ammonia nitrogen levels in water exert toxic effects on vital organs, such as the liver and kidneys (Figure 2), leading to the inactivation of antioxidant enzymes, such as SOD, CAT, and GPx, and subsequent increases in MDA content [40].

### 3.5. Changes in Gill and Liver Tissue Structure During Fish Transportation

**Figure 4:**
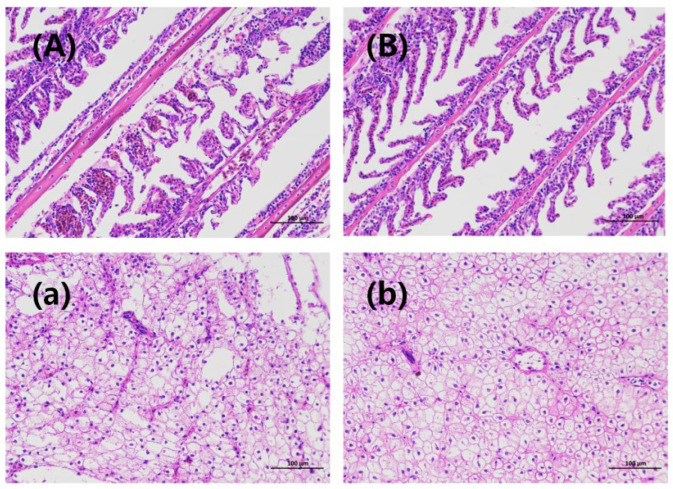
Changes in gill and liver tissue structure of Crucian carp after 48 h of transportation in fish bags. Images (**A**,**B**) show the structure of the gill tissue in the control group before and after 48 h of transportation, respectively. Images (**a**,**b**) show the structure of the liver tissue of the treatment group before transportation and after 48 h of transportation, respectively. Note: Representative images from three independent fish per group.

The gill tissue structure of the Crucian carp after 48 h of transportation is shown in Figure 4A,B. In the CG, most of the gill filaments exhibited severe bending, stacking, and entanglement. In addition, the epithelial cells on the gill lamellae were detached and showed significant vacuolation. The higher ammonia nitrogen concentration in the CG water induced more severe stress responses in Crucian carp, resulting in significant deformation of the gill tissue structure in this group. In contrast, the gill structure of the TG remained relatively distinct, with most gill filaments showing only mild bending and congestion of the gill lamellae. The degree of filament entanglement was less severe than that in the CG. This indicates that the ammonia-nitrogen-reducing biofilm in the TG partially mitigated the effects of ammonia-nitrogen stress on Crucian carp, resulting in milder gill tissue deformation. Ammonia-nitrogen exposure causes severe gill damage, including lamellar fusion, epithelial cell loss, and chlorine-secreting cell proliferation. Peng et al. [9] demonstrated that prolonged exposure to high ammonia nitrogen concentrations induces dissociation, disorganization, and rupture in gill tissues. These findings align with the structural alterations observed in the gills in this study.

Liver tissue structure of the Crucian carp after 48 h of transport is depicted in Figure 4a,b. Following HE staining, the nuclei appear dark blue, whereas the cytoplasm, myofibrils, and other structures exhibit a light pink hue. The remaining white areas primarily represent intercellular spaces [41]. As shown in Figure 4a, hepatocytes in the CG exhibited incomplete structures that were loose and disordered. Numerous cells ruptured, forming extensive, continuous vacuoles accompanied by nuclear dissolution and enlarged intercellular spaces. As shown in Figure 4b, hepatocyte structural integrity was reduced in the TG, with diminished nuclear clarity and an indistinct cell outline. However, no large vacuoles were observed, and the hepatocyte boundaries remained relatively clear.

Following ammonia-nitrogen treatment, black bass hepatocytes exhibited a more disordered arrangement, significant enlargement, increased nuclear fragmentation and vacuolarization, and marked expansion of the hepatic sinusoidal network [34]. In juvenile four-fingered threadfin bream, prolonged ammonia-nitrogen exposure progressively worsens hepatocyte vacuolation and nucleolar blurring [39]. Other studies have revealed partial detachment of respiratory epithelial cells in the gill lamellae, curled gill lamellae, and aggravated tissue congestion in juvenile striped seahorses subjected to ammonia-nitrogen stress [42]. These findings are consistent with the histological alterations observed in the gills and livers of the fish in the present study. During live transport in fish bags, the ammonia-nitrogen-reducing biofilm effectively mitigated ammonia-nitrogen toxicity to Crucian carp gills and liver in the TG by adsorbing and converting ammonia-nitrogen in water, thereby reducing its concentration. While histological observations revealed clear differences between CG and TG, we acknowledge that quantitative scoring and statistical analysis of tissue damage were not performed in this study. Nonetheless, the qualitative differences observed are consistent with the biochemical and physiological data, supporting the protective role of the biofilm.

### 3.6. Changes in Muscle Quality During Live Transport

#### 3.6.1. Changes in Drip Loss, Shear Force, Glycogen, Lactate, and pH

**Figure 5:**
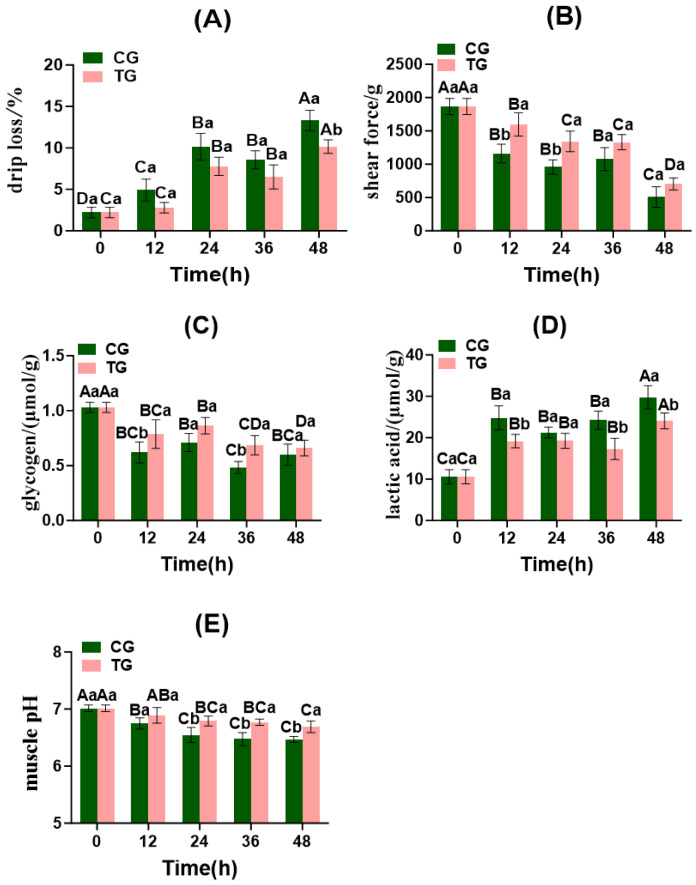
Changes in drip loss, shear force, glycogen, lactic acid, and pH value of Crucian carp muscle during transportation in fish bags. CG: control group; TG: treatment group. Note: Values are represented mean ± SD. For drip loss and shear force (**A**,**B**), *n* = 3 fish per group per time point. For glycogen, lactate, and pH (**C**–**E**), *n* = 10 fish per group per time point. Different capital letters indicate significant differences within the same group at different time points (*p* < 0.05), and different lowercase letters indicate significant differences among different groups at the same time point (*p* < 0.05).

As shown in Figure 5A,B, significant differences in muscle drip loss were observed between the CG and TG after 48 h of live transport (*p* < 0.05). During the early phase of live transport (0–24 h), the muscle drip loss in both the CG and TG showed a gradual increase. However, by 36 h of live transport, these values decreased to 8.57% and 6.50%, respectively. At 48 h of live transport, muscle drip loss reached its maximum values of 13.33% and 10.16% in the CG and TG, respectively. Muscle shear force differed significantly between the CG and TG at 12 and 24 h of transport (*p* < 0.05). At 48 h of live transport, the muscle shear force reached minimum values of 506.67 g and 703.33 g in the CG and TG, respectively.

As shown in Figure 5C–E, glycogen content differed significantly between the CG and TG at 12 and 36 h post-transport (*p* < 0.05). At 36 and 48 h post-live transport, glycogen content reached minimum values of 0.48 μmol/g and 0.66 μmol/g in the CG and TG, respectively. Muscle lactate levels in the CG and TG differed significantly at 12, 36, and 48 h of live transport (*p* < 0.05). At 48 h of live transport, muscle lactate levels reached maximum values of 29.77 μmol/g and 24.06 μmol/g in the CG and TG, respectively. The muscle pH in both the CG and TG exhibited a decreasing trend with prolonged survival time, showing significant differences at 24, 36, and 48 h of live transport (*p* < 0.05). The muscle pH in both groups reached its minimum value at 48 h of live transport.

These results indicate that transport stress significantly accelerates anaerobic glycolysis in fish muscle, leading to rapid glycogen depletion, substantial lactic acid accumulation, and cell apoptosis. This results in decreased muscle pH, accelerated protein denaturation, and myofibrillar structural damage, ultimately manifesting as reduced shear force and increased drip loss. Previous studies have demonstrated that energy metabolism disruption (Section 3.3.2) and lactic acid accumulation under stress are key factors in the deterioration of fish muscle quality. These processes weaken the muscle water-holding capacity and textural properties by lowering the pH and disrupting the spatial structure of myofibrillar proteins [43]. LDH in fish mediates redox reactions involving lactate during glycolysis and gluconeogenesis [44]. Consequently, increased LDH activity in Crucian carp elevates muscle lactate content (Section 3.3.2). In contrast, TG effectively maintained the structural stability of myofibrillar proteins by slowing glycogen depletion, inhibiting excessive lactate production, and delaying muscle acidification. This significantly improved the muscle water-holding capacity and textural properties during transport, consistent with prior research showing that alleviating transport stress effectively enhances fish muscle quality [45]. Muscle lactate levels in the TG remained consistently lower than those in the CG, whereas muscle glycogen content and pH values were higher in the TG. This indicates that the ammonia-nitrogen-reducing biofilm in the TG effectively controlled ammonia-nitrogen concentrations in the water, thereby reducing stress responses and energy expenditure in Crucian carp, ultimately minimizing muscle tissue damage.

#### 3.6.2. Changes in Muscle Color

As shown in Table 2, significant alterations in muscle color occurred during live transport in fish bags, primarily manifested as reduced brightness and whiteness, as well as diminished color stability. Both CG and TG exhibited declining trends in L* and W values, reaching their lowest points at 48 h, indicating that transport stress caused the fish meat to gradually darken. The CG demonstrated a more pronounced decrease in L* and W values, which were significantly lower than those at the start of transport (*p* < 0.05). In contrast, the TG maintained relatively higher L* and W values during the mid-to-late period (24–36 h), suggesting that the treatment measures partially delayed the deterioration of muscle brightness and whiteness. The a* value reached its peak at 24 h and lowest point at 48 h. The b* value exhibited temporal variation in both groups: the CG showed an overall trend of initial decline followed by recovery, whereas the TG exhibited a significant increase in b* at 36 h, which subsequently decreased in the later transport phase. Previous studies have indicated that transport or environmental stress can lead to glycogen depletion, lactic acid accumulation, and pH decline in the muscle, thereby affecting muscle pigment stability and protein structure, resulting in reduced L* and W values [46]. Effective stress mitigation helps maintain the color and appearance of fish meat, consistent with the present findings. As survival time increased, the W values gradually decreased in both the CG and TG. Nitrification by the ammonia-nitrogen-reducing biofilm in the TG mitigated ammonia-nitrogen stress responses in crucian carp, resulting in less severe muscle damage in this group.

#### 3.6.3. Changes in ATP and Its Metabolically Related Products

As shown in Table 3, muscle ATP content in both the CG and TG reached its minimum after 24 h of live transport. Muscle ADP content in the CG gradually decreased during the early phase of live transport before rising to 20.51 mg/100 g. Muscle ADP content in the TG reached its lowest point at 11.84 mg/100 g after 24 h of live transport. Muscle AMP content in the CG peaked at 7.81 mg/100 g after 24 h of live transport; muscle AMP content in the TG reached its highest level at 6.72 mg/100 g after 12 h of live transport. IMP levels in both CG and TG showed an initial increase followed by a decline. At 24 h of live transport, the CG muscle IMP reached its maximum at 211.54 mg/100 g; at 36 h of live transport, the TG muscle IMP peaked at 214.95 mg/100 g. Bitter compounds HxR and Hx in the CG muscle gradually accumulated, with Hx reaching a maximum of 9.81 mg/100 g at 36 h of live transport. In the TG, HxR gradually accumulated with extended live transport time, peaking at 7.72 mg/100 g at 48 h. The freshness value K of CG and TG muscle gradually increased from 4.41% at 0 h to 6.62% and 5.96% at 48 h of live transport.

As live transport duration increased, muscle freshness declined. Following ATP degradation, AMP was initially formed, which was subsequently converted to IMP. IMP was further degraded into HxR and Hx over extended live transport periods [47]. IMP is a key umami-contributing compound whose concentration rapidly increases post-stress, while the ultimately accumulated Hx is considered a major factor causing flavor deterioration and bitterness [48]. Abe et al. [49] reported that carp subjected to pre-slaughter stress (e.g., crowding) exhibited significantly faster IMP accumulation in muscle tissue compared to fish slaughtered under calm conditions. This may result from stress accelerating energy consumption and catabolic metabolism. Fan et al. [12] found that adding 0.4 mg/L ATP to water modulated the immune defense capacity and reduced apoptosis levels in Pacific white shrimp under cold stress. Postmortem ATP degradation rates typically far exceed production rates, resulting in the rapid depletion of ATP content. Higher K values indicate poorer muscle freshness [50]. During transport, the K values in the CG muscles consistently exceeded those in the TG, demonstrating that the ammonia-nitrogen-degrading biofilm effectively reduced ammonia-nitrogen levels in water during live fish transport, enhanced Crucian carp muscle quality, and mitigated stress responses.

#### 3.6.4. Changes in Muscle Redox Indicators and ROS Staining

**Figure 6:**
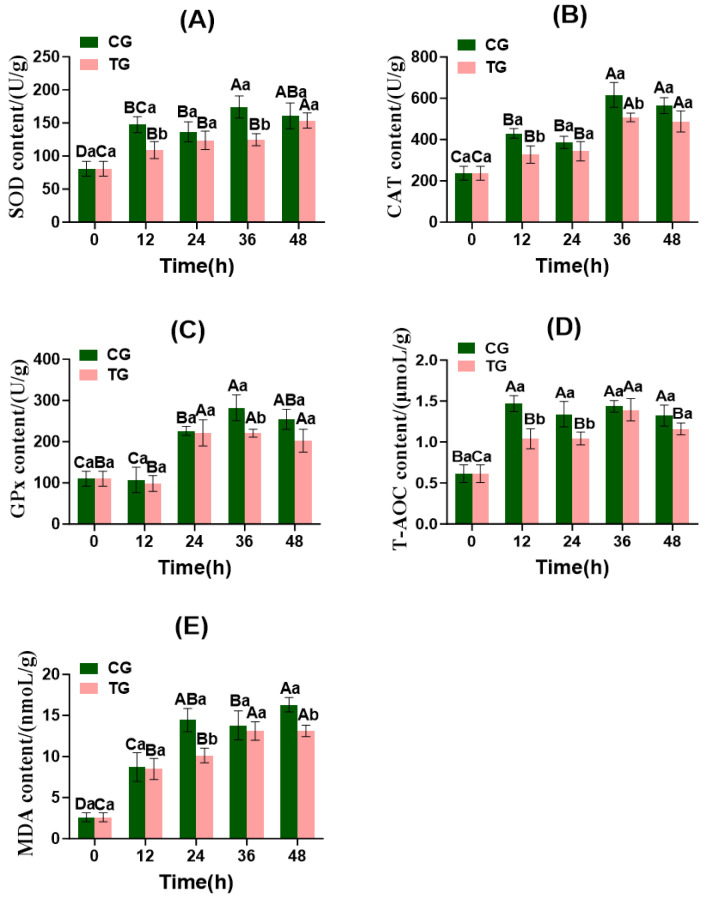
Changes in redox indicators of common carp muscle during transportation in fish bags. CG: control group; TG: treatment group. (**A**) SOD, superoxide dismutase; (**B**) CAT, catalase; (**C**) GPx, glutathione peroxidase; (**D**) T-AOC, total antioxidant capacity; (**E**) MDA, mal. Note: Values are represented mean ± SD of three independent replicates (*n* = 3) per group. Different capital letters indicate significant differences within the same group at different time points (*p* < 0.05), and different lowercase letters indicate significant differences among different groups at the same time point (*p* < 0.05).

As shown in Figure 6, significant differences in redox indicators were observed between the CG and TG (*p* < 0.05). During live transport in fish bags, SOD, CAT, and GPx activities in Crucian carp muscle increased throughout the process, with antioxidant enzyme activities generally being higher in the CG muscles than in the TG. This suggests that stress leads to a substantial accumulation of free radicals in fish, triggering oxidative stress responses. The application of ammonia-nitrogen-reducing biofilms partially alleviated stress responses in Crucian carp, mitigating free radical damage to the body. Srikanth et al. [44] observed that antioxidant enzyme activity increased with prolonged stress duration. CAT activity in both the TG and CG decreased after 24 h of live transport, whereas T-AOC content abruptly increased at 12 h and declined at 24 h. This may occur because, during the early transport phase, muscle tissue primarily relies on the antioxidant enzyme system to counteract oxidative stress induced by environmental stressors. Enhanced antioxidant enzyme activity leads to elevated T-AOC content. SOD and CAT changes in the CG and TG showed similar trends, indicating a positive correlation between the two antioxidant enzymes [36]. The T-AOC and GPx levels in the crucian carp muscle peaked and then gradually declined, suggesting a dynamic equilibrium between oxidative stress and the antioxidant defense system following ammonia nitrogen stress, prompting the activation of antioxidant enzyme systems to scavenge reactive oxygen species [37]. As the antioxidant enzyme system continues to operate, its enzymatic activity gradually diminishes, leading to decreased total antioxidant capacity and GPx levels. MDA content in muscle tissue increased in both the CG and TG, indicating heightened lipid peroxidation in Crucian carp muscle under transport stress and ammonia nitrogen stress.

**Figure 7:**
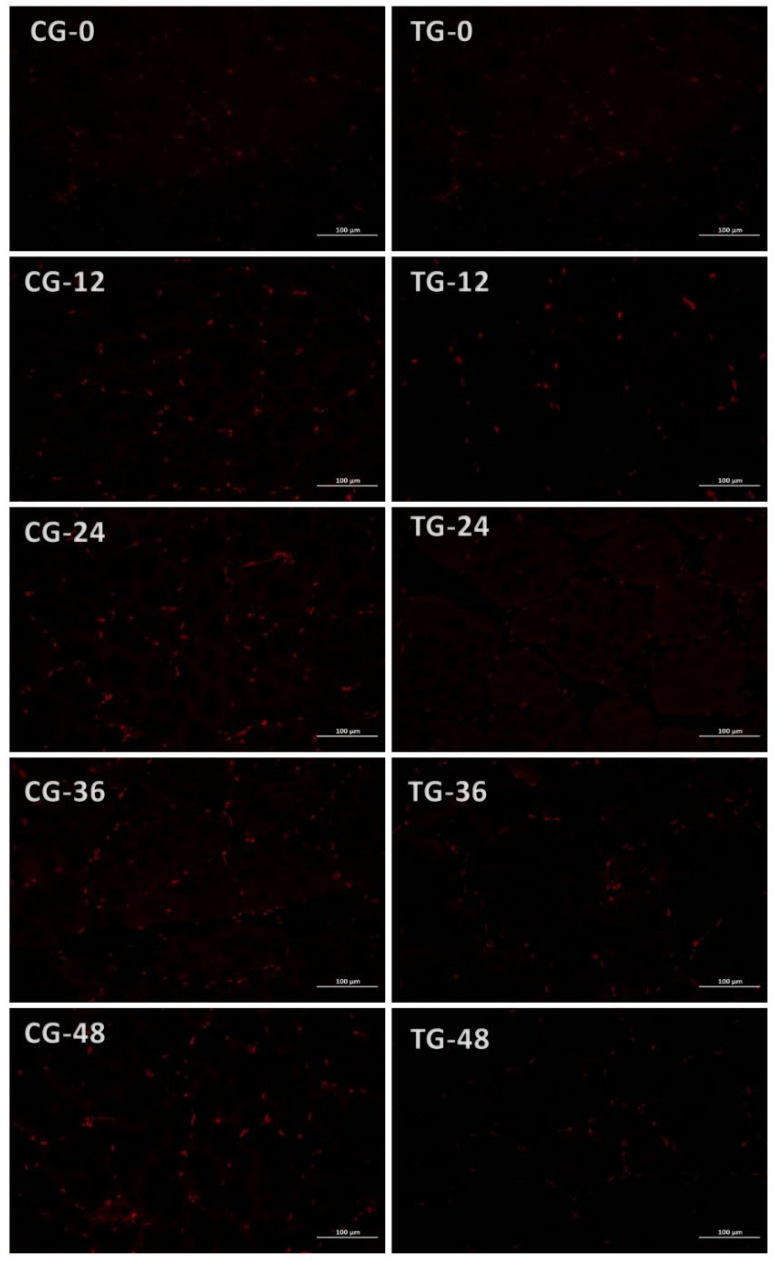
Changes in ROS staining in the muscles of Crucian carp during transportation in fish bags. CG: control group; TG: treatment group. The denoted number indicates the transportation time (h). Note: Representative fluorescence images from three independent fish per group (*n* = 3).

As shown in Figure 7, during live transport in fish bags, red indicates ROS-stained areas in the Crucian carp muscle. ROS levels progressively increased in both the CG and TG, with the CG consistently exhibiting higher ROS content than the TG. This resulted from oxygen depletion within the bags, while the TG water contained lower ammonia nitrogen levels than the CG, indicating milder ammonia nitrogen stress in the former. Following exposure to ammonia nitrogen, previous studies on Crucian carp and its hybrid varieties have shown that the activities of enzymes such as SOD in tissues like the liver exhibit an initial increase followed by a decrease [37].

#### 3.6.5. Apoptotic Changes in Muscle Cells

**Figure 8:**
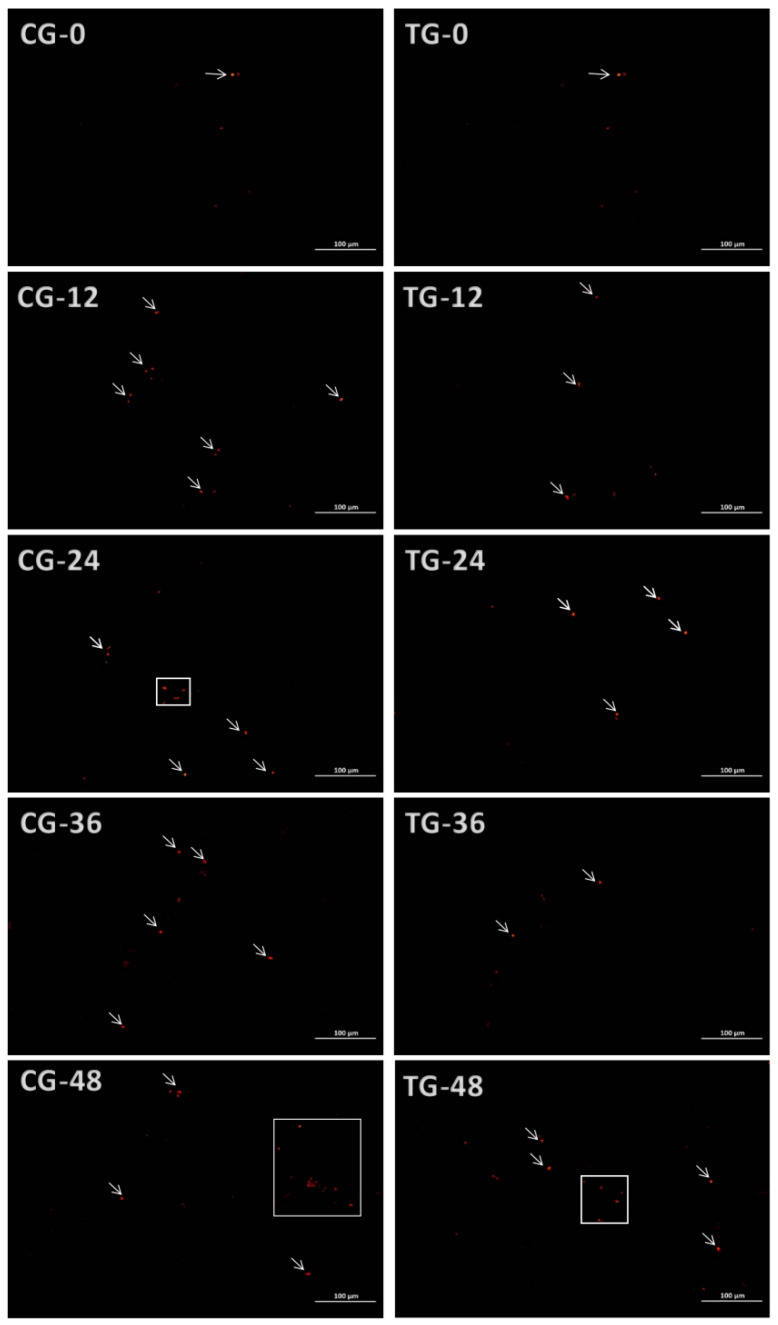
Changes in TUNEL staining in muscle cells of Crucian carp during transportation in fish bags. CG: control group; TG: treatment group. The denoted number indicates transportation time (h). Note: Representative fluorescence images from three independent fish per group (*n* = 3). White arrowheads indicate representative apoptotic cells. White squares indicate representative areas of apoptosis.

As shown in Figure 8, TUNEL staining typically employs fluorescent labeling during live transport in fish bags. Normal cells exhibit no fluorescence, while apoptotic cells display red fluorescence. As live transport duration increases, stress responses and metabolic processes in fish lead to the production of substances such as ammonia nitrogen. Ammonia nitrogen inhibits the expression of antioxidant enzyme-related genes in fish, increasing the rate of apoptosis [51,52]. At 12, 24, 36, and 48 h of live transport, both the CG and TG exhibited higher levels of apoptosis than at the 0 h baseline. TUNEL-based detection revealed a slower increase in apoptotic cell numbers in TG Crucian carp muscle cells, whereas varying degrees of apoptosis were detected in the CG. Apoptotic cell counts were generally higher in the CG than in the TG; this may be attributed to the ammonia-nitrogen-reducing biofilm in the TG, which mitigated stress responses through nitrification-induced ammonia reduction in water. TUNEL staining revealed significantly increased DNA fragmentation in muscle cells under stress conditions, leading to a higher number of positive cells.

Upregulation of *caspase-3* gene expression is crucial for apoptosis, and the expression levels of apoptosis-related proteins, such as *bcl-2* and *bax*, also change accordingly. These alterations collectively regulate apoptosis [53]. Upon apoptotic stimulation (e.g., ammonia-induced oxidative stress), pro-apoptotic proteins such as Bax translocate to the mitochondria and increase outer membrane permeability, leading to the release of cytochrome c into the cytosol. Cytochrome c then binds to Apaf-1 to form the apoptosome, which activates *caspase-9*. *Caspase-9* in turn cleaves and activates the executioner *caspase-3*, ultimately resulting in DNA fragmentation [53,54]. Under high-temperature conditions, apoptotic signals were detected in the dorsal muscles of Nile tilapia, indicating that environmental stress induces muscle cell apoptosis [55]. Studies suggest that *bcl-2* family proteins trigger apoptosis by regulating the permeability of the outer mitochondrial membrane [54]. *Caspase-3* activity progressively increased with extended live transport duration, consistent with the muscle cell apoptosis trend observed in TUNEL assays. Ammonia nitrogen stress also induces oxidative stress in crucian carp, leading to decreased antioxidant enzyme (SOD, CAT, and GPx) activity, which exacerbates apoptosis.

#### 3.6.6. Changes in Muscle Tissue Structure

**Figure 9:**
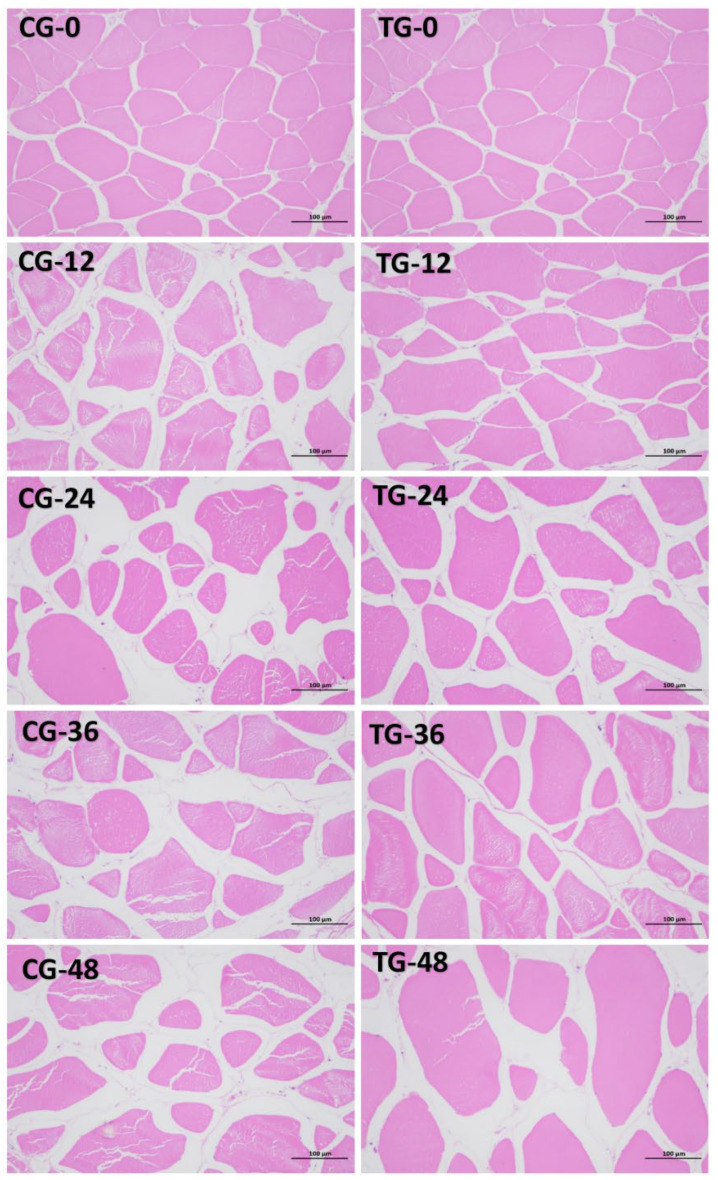
Changes in the muscle tissue structure of Crucian carp during transportation in fish bags. CG: control group; TG: treatment group. The denoted number indicates transportation time (h). Note: Values are represented mean ± SD of three independent replicates (*n* = 3) per group.

As shown in Figure 9, during live transport in fish bags, normal crucian carp muscle cells exhibited a spindle-shaped morphology with tightly arranged muscle fibers and no signs of bending. After 12 h of live transport in fish bags, the CG showed significant muscle tissue damage. Fiber bundles lost their original compact structure; muscle fibers ruptured, and interstitial spaces widened markedly. In the TG, muscle fibers in some areas exhibited twisted deformations, with widened gaps between muscle bundles and loose arrangements in a small number of fibers. At 24 and 36 h post-transport, the CG showed severely twisted deformation damage, disordered muscle fiber arrangement, increased fiber breaks, larger gaps between bundles, and intensified vacuolation. The TG also exhibited progressively widening gaps between muscle fibers, with subsequent ruptures and partial cellular fragmentation. After 48 h of live transport, muscle tissue damage in the CG intensified further. Deeper muscle cell degeneration, loss of normal morphology, and progressive tissue disintegration were observed. The TG exhibited larger intercellular gaps but less severe structural damage than the CG. Peng et al. [56] observed that prolonged viability transport increased intermuscular gaps and disrupted muscle fiber structure in Crucian carp, consistent with the present findings. The results from Section 3.6.4 indicate that extended stress duration and accumulated ammonia nitrogen elevate oxidative stress levels in Crucian carp, leading to increased reactive oxygen species (ROS) content. ROS accumulation damages muscle cell membranes, proteins, and lipids, thereby disrupting the structural integrity of muscle tissue. The ammonia-nitrogen-reducing biofilm lowers ammonia-nitrogen levels in water through nitrification, thereby reducing stress responses in fish and improving Crucian carp muscle quality.

## 4. Conclusions

This study demonstrates that the application of ammonia-nitrogen-reducing biofilms in fish bag transport systems can mitigate water quality, attenuate stress responses, and help preserve muscle quality in Crucian carp during bag transport. Through nitrification, the biofilms significantly reduced ammonia-nitrogen concentrations in transport water (with a maximum decrease of 31.2% over 72 h) and slowed pH decline and dissolved oxygen consumption. This helps maintain relative stability in transport water. Compared to the CG, the TG showed significantly lower stress indicators in Crucian carp serum, including cortisol, glucose, and lactate dehydrogenase. Structural damage to gill filaments and hepatocytes was markedly reduced, and the 72 h survival rate increased from 0% to 20%, demonstrating excellent stress resistance and protective effects. The TG muscles exhibited superior water-holding capacity, textural properties, and freshness indicators. These were shown as reduced drip loss, increased shear force, slowed glycogen consumption, decreased lactic acid accumulation, delayed ATP degradation rate, and significantly lower K values compared to the CG. This indicates that biofilms can delay muscle quality deterioration by alleviating stress.

The ammonia-nitrogen-reducing biofilm demonstrates the dual functions in water purification and physiological protection during live fish transport. These findings highlight its promising application potential. This study provides theoretical foundations and technical references for advancing eco-friendly, low-stress live fish transportation technologies.

## Figures and Tables

**Table 1:** The influence of different loading amounts on water quality indicators within 72 h of fish transportation at 25 °C.

Water Quality Index	Load/Piece	0 h	12 h	24 h	36 h	48 h	60 h	72 h
pH	0	7.95 ± 0.07 ^a^	7.42 ± 0.04 ^a^	7.66 ± 0.03 ^a^	7.70 ± 0.02 ^a^	7.57 ± 0.05 ^a^	7.22 ± 0.03 ^a^	7.28 ± 0.03 ^a^
	2	7.95 ± 0.07 ^a^	7.34 ± 0.04 ^a^	7.68 ± 0.06 ^a^	7.11 ± 0.02 ^b^	7.04 ± 0.04 ^b^	7.07 ± 0.05 ^b^	6.91 ± 0.03 ^b^
	5	7.95 ± 0.07 ^a^	7.19 ± 0.04 ^a^	7.43 ± 0.03 ^a^	7.23 ± 0.06 ^b^	7.16 ± 0.02 ^b^	6.94 ± 0.03 ^b^	6.92 ± 0.04 ^b^
dissolved oxygen (mg/L)	0	5.80 ± 0.10 ^a^	6.70 ± 0.20 ^b^	7.73 ± 0.21 ^a^	6.47 ± 0.32 ^a^	5.67 ± 0.15 ^a^	5.30 ± 0.26 ^a^	4.63 ± 0.25 ^a^
	2	5.80 ± 0.10 ^a^	6.47 ± 0.21 ^b^	6.53 ± 0.15 ^b^	5.83 ± 0.25 ^ab^	4.73 ± 0.50 ^b^	4.67 ± 0.15 ^b^	4.23 ± 0.35 ^a^
	5	5.80 ± 0.10 ^a^	7.40 ± 0.30 ^a^	6.57 ± 0.31 ^b^	5.30 ± 0.20 ^b^	5.23 ± 0.15 ^a^	4.60 ± 0.26 ^b^	3.77 ± 0.15 ^b^
Ammonia nitrogen (mg/L)	0	0.07 ± 0.01 ^a^	8.49 ± 1.01 ^a^	18.35 ± 0.52 ^a^	26.48 ± 0.43 ^a^	32.45 ± 0.32 ^a^	38.27 ± 0.34 ^a^	46.64 ± 0.87 ^a^
	2	0.07 ± 0.01 ^a^	7.25 ± 0.98 ^b^	13.88 ± 0.57 ^b^	20.65 ± 0.57 ^b^	23.09 ± 1.15 ^c^	32.50 ± 0.63 ^b^	37.08 ± 0.12 ^b^
	5	0.07 ± 0.01 ^a^	6.52 ± 0.21 ^c^	9.11 ± 0.26 ^c^	18.32 ± 1.02 ^c^	27.47 ± 0.06 ^b^	29.42 ± 0.65 ^c^	35.69 ± 0.48 ^b^
nitrite (mg/L)	0	0.07 ± 0.01 ^a^	0.14 ± 0.01 ^b^	0.21 ± 0.01 ^b^	0.25 ± 0.01 ^c^	0.32 ± 0.01 ^b^	0.36 ± 0.04 ^c^	0.45 ± 0.02 ^c^
	2	0.07 ± 0.01 ^a^	0.18 ± 0.03 ^b^	0.28 ± 0.01 ^b^	0.44 ± 0.02 ^b^	0.50 ± 0.04 ^ab^	0.53 ± 0.02 ^b^	0.69 ± 0.01 ^b^
	5	0.07 ± 0.01 ^a^	0.22 ± 0.02 ^a^	0.37 ± 0.02 ^a^	0.53 ± 0.01 ^a^	0.58 ± 0.01 ^a^	0.66 ± 0.01 ^a^	0.75 ± 0.03 ^a^

Note: Values are represented mean ± SD of three independent replicates (*n* = 3) per group. Different lowercase letters in the same column indicate significant differences (*p* < 0.05).

**Table 2:** Changes in muscle coloration during transportation in fish bags.

Contratest	Time/h	L*	a*	b*	W
CG	0	49.23 ± 0.89 ^ab^	2.05 ± 1.00 ^a^	5.43 ± 1.00 ^a^	48.88 ± 0.86 ^ab^
	12	50.08 ± 3.14 ^a^	0.71 ± 0.28 ^b^	1.79 ± 1.36 ^c^	50.02 ± 3.08 ^a^
	24	46.18 ± 1.62 ^abc^	2.41 ± 2.91 ^a^	3.82 ± 2.24 ^b^	45.91 ± 1.80 ^abc^
	36	44.55 ± 1.46 ^bc^	−0.01 ± 1.14 ^c^	3.87 ± 1.76 ^b^	44.39 ± 1.58 ^bc^
	48	42.07 ± 1.03 ^c^	1.92 ± 0.90 ^ab^	5.16 ± 0.87 ^a^	41.80 ± 0.97 ^c^
TG	0	49.23 ± 0.89 ^ab^	2.05 ± 1.00 ^a^	5.43 ± 1.00 ^ab^	48.88 ± 0.86 ^ab^
	12	50.85 ± 1.57 ^a^	0.74 ± 0.83 ^ab^	3.15 ± 1.80 ^b^	50.72 ± 1.60 ^a^
	24	47.31 ± 0.76 ^b^	0.29 ± 0.15 ^ab^	3.53 ± 0.37 ^b^	47.18 ± 0.78 ^b^
	36	47.16 ± 1.07 ^b^	1.60 ± 0.82 ^a^	7.23 ± 0.42 ^a^	46.64 ± 1.03 ^b^
	48	43.81 ± 0.34 ^c^	−0.39 ± 0.08 ^b^	2.83 ± 0.39 ^b^	43.73 ± 0.32 ^c^

Note: CG: control group; TG: treatment group. L*: brightness value of color, a*: red green degree value, b*: yellow blue chromaticity value, W: the degree of whiteness. Values are represented mean ± SD of ten independent replicates (*n* = 10) per group. Different lowercase letters in the same column indicate significant differences (*p* < 0.05).

**Table 3:** Changes in muscle ATP and metabolic products during transportation in fish bags.

Contratest	Time (h)	Content (mg/100 g)						K (%)
		ATP	ADP	AMP	IMP	HxR	Hx	
CG	0	6.35 ± 2.24 ^a^	19.53 ± 2.07 ^ab^	4.84 ± 0.85 ^c^	190.54 ± 8.87 ^c^	3.84 ± 2.02 ^c^	7.30 ± 1.21 ^c^	4.41 ± 0.77 ^b^
	12	5.84 ± 0.17 ^a^	17.42 ± 0.51 ^bc^	5.27 ± 1.77 ^bc^	203.47 ± 13.04 ^b^	7.85 ± 1.07 ^ab^	8.51 ± 0.52 ^b^	6.59 ± 0.93 ^a^
	24	2.38 ± 1.54 ^c^	16.25 ± 1.47 ^c^	5.88 ± 1.18 ^b^	211.54 ± 9.24 ^a^	6.94 ± 0.88 ^b^	9.57 ± 0.61 ^a^	6.54 ± 0.54 ^a^
	36	4.58 ± 0.55 ^b^	18.36 ± 2.24 ^b^	6.32 ± 0.80 ^b^	194.73 ± 8.35 ^c^	8.56 ± 1.85 ^a^	9.81 ± 1.23 ^a^	7.58 ± 1.21 ^a^
	48	2.45 ± 2.30 ^c^	20.51 ± 2.32 ^a^	7.81 ± 1.49 ^a^	196.38 ± 12.02 ^c^	8.53 ± 2.54 ^a^	7.85 ± 1.02 ^bc^	6.62 ± 0.62 ^a^
TG	0	6.35 ± 2.24 ^a^	19.53 ± 2.07 ^a^	4.48 ± 0.85 ^c^	190.54 ± 8.87 ^d^	3.84 ± 2.02 ^c^	7.30 ± 1.21 ^ab^	4.41 ± 1.12 ^b^
	12	3.59 ± 2.41 ^c^	16.24 ± 2.17 ^b^	6.72 ± 2.04 ^a^	198.54 ± 9.57 ^c^	5.84 ± 0.18 ^b^	7.15 ± 0.96 ^ab^	5.46 ± 0.75 ^ab^
	24	2.32 ± 0.53 ^d^	11.84 ± 1.20 ^c^	5.64 ± 1.47 ^b^	207.87 ± 5.51 ^b^	6.65 ± 1.25 ^ab^	5.39 ± 0.87 ^c^	4.98 ± 0.31 ^ab^
	36	4.94 ± 1.27 ^b^	19.35 ± 0.84 ^a^	2.87 ± 0.94 ^d^	214.95 ± 5.25 ^a^	5.39 ± 2.14 ^b^	7.64 ± 0.36 ^a^	5.07 ± 0.47 ^ab^
	48	3.25 ± 1.34 ^c^	17.55 ± 2.17 ^b^	5.89 ± 2.45 ^b^	201.52 ± 8.64 ^c^	7.72 ± 1.76 ^a^	6.81 ± 0.34 ^b^	5.96 ± 1.01 ^a^

Note: CG: control group; TG: treatment group. ATP: 5′-adenosine triphosphate, ADP: 5′-adenosine diphosphate, AMP: 5′-adenosine monophosphate, IMP: 5′-inosinic acid, HxR: inosine, Hx: hypoxanthine, K: muscle freshness value. Values are represented mean ± SD of three independent replicates (*n* = 3) per group. Different lowercase letters in the same column indicate significant differences (*p* < 0.05).

## Data Availability

The original contributions presented in the study are included in the article/Supplementary Materials. Further inquiries can be directed to the corresponding author.

## Supplementary Materials

The following supporting information can be downloaded at: https://www.mdpi.com/article/10.3390/foods15071189/s1, Table S1. Effect of different tablet dosage and temperature on ammonia nitrogen content of water body.
