# Supplementary material for: Effects of Ammonia-Nitrogen-Reducing Biofilm on Stress Responses and Muscle Quality in Crucian Carp During Transportation
Source: Foods. 2026 Apr 1;15(7):1189. doi: 10.3390/foods15071189 (PMC13072757; doi:10.3390/foods15071189)
Supplement: Supplementary file 1 [file foods-15-01189-s001.zip › foods-4164408-supplementary.pdf]

Supplement Table S1 Effect of different tablet dosage and temperature on ammonia nitrogen content of water body

Load/piece	temperature	6 h	12 h	24 h	36 h	48 h	60 h	72 h
0.5	4 ℃	49.65 ± 0.27^{a}	49.32 ± 0.18^{a}	48.35 ± 0.78^{a}	48.69 ± 0.54^{a}	48.51 ± 0.87^{a}	47.75 ± 0.51^{a}	46.81 ± 0.76^{a}
	10 ℃	48.53 ± 1.22^{ab}	48.55 ± 0.81^{a}	46.80 ± 0.64^{b}	46.92 ± 0.95^{b}	46.74 ± 0.09^{b}	45.35 ± 0.67^{b}	43.97 ± 0.81^{b}
	25 ℃	47.98 ± 0.75^{b}	44.72 ± 0.39^{b}	44.30 ± 0.55^{c}	43.27 ± 0.34^{c}	42.65 ± 0.73^{c}	41.79 ± 0.14^{c}	41.32 ± 0.78^{c}
1.0	4 ℃	48.75 ± 0.83^{a}	48.99 ± 0.85^{a}	48.37 ± 0.76^{a}	47.52 ± 0.34^{a}	46.03 ± 0.52^{a}	45.46 ± 0.75^{a}	44.21 ± 0.65^{a}
	10 ℃	47.62 ± 0.92^{a}	46.28 ± 0.71^{b}	45.30 ± 0.27^{b}	45.58 ± 0.08^{b}	44.99 ± 0.36^{a}	43.10 ± 0.31^{b}	42.57 ± 0.91^{a}
	25 ℃	47.13 ± 0.79^{a}	44.44 ± 0.57^{c}	43.31 ± 0.86^{c}	42.36 ± 0.35^{c}	41.39 ± 0.41^{b}	40.45 ± 0.53^{c}	39.56 ± 0.38^{b}
1.5	4 ℃	49.52 ± 0.56^{a}	48.24 ± 0.37^{a}	47.20 ± 0.21^{a}	46.53 ± 0.74^{a}	46.13 ± 0.42^{a}	44.32 ± 0.35^{a}	43.59 ± 0.61^{a}
	10 ℃	48.44 ± 0.44^{a}	47.21 ± 0.49^{a}	46.56 ± 0.36^{a}	43.86 ± 0.81^{b}	43.43 ± 0.75^{b}	41.28 ± 0.54^{b}	40.86 ± 0.56^{b}
	25 ℃	46.81 ± 0.71^{b}	45.84 ± 0.81^{b}	41.96 ± 0.57^{b}	40.31 ± 0.72^{c}	38.45 ± 0.61^{c}	37.64 ± 0.57^{c}	37.22 ± 0.64^{c}
2.0	4 ℃	48.54 ± 0.11^{a}	46.79 ± 0.75^{a}	45.74 ± 0.44^{a}	45.31 ± 0.68^{a}	44.50 ± 0.73^{a}	42.26 ± 0.94^{a}	40.79 ± 0.61^{a}
	10 ℃	46.83 ± 0.77^{b}	44.81 ± 0.45^{b}	45.37 ± 0.69^{a}	42.09 ± 0.72^{b}	40.03 ± 0.91^{b}	38.40 ± 0.87^{b}	36.05 ± 0.35^{b}
	25 ℃	46.25 ± 0.79^{b}	45.27 ± 0.67^{ab}	42.35 ± 0.21^{b}	38.15 ± 0.17^{c}	37.21 ± 0.59^{c}	36.95 ± 0.19^{b}	34.57 ± 0.44^{c}
2.5	4 ℃	48.21 ± 0.86^{a}	45.91 ± 0.36^{a}	45.86 ± 0.53^{a}	44.13 ± 0.46^{a}	43.60 ± 0.57^{a}	41.19 ± 0.65^{a}	39.83 ± 0.38^{a}
	10 ℃	47.88 ± 0.24^{a}	45.19 ± 0.65^{a}	44.28 ± 0.47^{a}	42.78 ± 0.74^{a}	40.88 ± 0.36^{b}	38.52 ± 0.42^{b}	37.95 ± 0.27^{b}
	25 ℃	47.64 ± 0.32^{a}	43.23 ± 0.24^{b}	40.64 ± 0.15^{b}	37.48 ± 0.39^{b}	36.53 ± 0.68^{c}	34.95 ± 0.77^{c}	32.45 ± 0.69^{c}

Note: Different lowercase letters in the same column indicate significant differences ($p<0.05$)
